# Searching therapeutic strategy of new coronavirus pneumonia from angiotensin-converting enzyme 2: the target of COVID-19 and SARS-CoV

**DOI:** 10.1007/s10096-020-03883-y

**Published:** 2020-04-13

**Authors:** Shu-ren Li, Zi-jian Tang, Zai-han Li, Xuan Liu

**Affiliations:** 1 Hebei General Hospital, Shijiazhuang, 050051 Hebei China; 2 Hebei General Hospital, Graduate School of Hebei Medical University, Shijiazhuang, 050000 China; 3 Suzhou University, Suzhou, 215000 Jiangsu China; 4 The Fourth Hospital of Hebei Medical University, Shijiazhuang, 050000 China

**Keywords:** New coronavirus (COVID-19), Angiotensin-converting enzyme 2 (ACE2), SARS coronavirus (SARS-CoV)

## Abstract

Since December 2019, the infection of the new coronavirus (COVID-19) caused an outbreak of new coronavirus pneumonia in Wuhan, China, and caused great public concern. Both COVID-19 and SARS-CoV belong to the coronavirus family and both invade target cells through ACE2. An in-depth understanding of ACE2 and a series of physiological and physiological changes caused by the virus invading the human body may help to discover and explain the corresponding clinical phenomena and then deal with them timely. In addition, ACE2 is a potential therapeutic target. This article will summarize the role of ACE2 in multiple organ damage caused by COVID-19 and SARS-CoV, targeted blocking drugs against ACE2, and drugs that inhibit inflammation in order to provide the basis for subsequent related research, diagnosis and treatment, and drug development.

A prerequisite for coronavirus infection is its entry into host cells. In this process, spike protein (S protein) recognizes host cell receptors and induces fusion of viral and cell membranes. XU et al. [1] found that the S protein of COVID-19 is similar to the S protein of SARS coronavirus (SARS-CoV) through biological analysis. It can also interact with the ACE2 protein molecule on the surface of host cells through S protein to infect epithelial cells of the host. Therefore, ACE2 molecule is the key molecule for COVID-19 infection, and it may affect the process of COVID-19 infection of human cells by binding to ACE2 molecule. In addition, Shi Zhengli’s team from Wuhan Virus Research Institute published a paper [2], reporting that ACE2 is an essential protein for COVID-19 infected cells. This article will review the characteristics of the protein and target organ damage and therapeutic drugs.

## Angiotensin-converting enzyme 2 (ACE2)

Renin-angiotensin system (RAS) is an important neuroendocrine system in the human body, it including the classical angiotensin converting enzyme (ACE) -angiotensin II (AngII) -angiotensin II type 1 receptor (AT1R) axis, and it also includes thoese congeners of ACE that was named angiotensin-converting enzyme 2 (ACE2), angiotensin 1–7 [Ang-(1-7) and its receptor Mas, etc. composed ACE2-Ang-(1-7) -Mas axis. In 2000, researchers found ACE2 in human heart left ventricle cDNA library prepared from explanted hearts of heart transplant recipients and human lymphoma cDNA library [3]. Like ACE, ACE2 belongs to the zinc metalloproteinase family. The sequence identity between ACE2 and ACE is 42%. The ACE2 protein is 805 amino acids in length and is encoded by the ACE2 gene located on chromosome Xp22. It consists of 4 parts, namely, N-terminal signal peptide, catalytic extracellular domain, transmembrane domain, and C-terminal intracellular domain. ACE2 is a type 1 membrane protein with a catalytic domain on the extracellular surface. ACE2 hydrolyzes the carboxy-terminal leucine from AngI to produce the non-peptide Ang-(1-9), which can be converted into heptapeptide by ACE and other peptidases [4]. In addition, ACE2 can directly degrade AngII to Ang-(1-7). Ang-(1-7) acts on Mas receptors to relax blood vessels and anti-proliferative and anti-oxidative stress [5]. The ACE2-Ang-(1-7)-Mas axis formed by the participation of Ang-(1-7) can antagonize the ACE-AngII-AT1R axis, and the two together maintain the balance of the body. Coronavirus specifically binds to which part of ACE2. Scholars analyzed the protein crystals of testicular ACE2 and Drosophila ACE2 homologs using sequence similarity between ACE2 subtypes and found that the enzyme catalytic region of ACE2 is located in a deep groove at the top of extracellular proteins. The pimple surrounding this deep groove is negatively charged and may have the ability to bind to the positively charged region of the S protein; several small hydrophobic regions formed by hydrophobic residues around the pimple that are close to the negative charge may also bind to the S protein [6].

## Target organ damage and abnormal coagulation

### Heart injury

ACE2 is highly expressed in the heart, which also provides the necessary receptors for the virus to invade the heart. Oudit et al. [7] found that mice infected with SARS-CoV can cause ACE2-dependent myocardial infection and the expression of ACE2 decreased significantly, confirming the important role of ACE2 in mediating cardiac SARS-CoV infection. In addition, during the SARS outbreak in Toronto, SARS-CoV virus RNA was detected in 35% (7/20) of autopsy heart samples of patients who died from SARS. Macrophage-specific staining showed a marked increase in macrophage infiltration in patients with SARS-CoV in the heart and evidence of myocardial injury. The presence of SARS-CoV in the heart is also associated with a significant decrease in ACE2 protein expression. This will cause an increase in AngII, the increased AngII have an importent role in the pathophysiology of cardiovascular disease by means of regulates the growth of cardiomyocytes, affect the intercellular and intracellular signaling mechanisms,decrease the intercellular communication and inhibit body immunity system,cause lipid peroxidation, and insulin resistance [8]. Antoniak et al. [9] found that after infusion of AngII into experimental WT mice for 28 days resulted in the mice aortic remodeling, accompanied by increased myocardial thickness and fibrosis. This eventually leads to cardiac hypertrophy, and coagulation associated with cardiac fibrosis and inflammation is activated. In addition, Ang-(1-7) will be less, and its cardiovascular protection will be weaker or even disappear. Therefore, SARS-CoV can mediate myocardial inflammation and myocardial ACE2 system downregulation-related damage. This may be the cause of myocardial dysfunction and adverse cardiac outcomes in patients with SARS. The mechanism of COVID-19 invasion of cells is roughly the same as SARS, and COVID-19 may cause heart damage through similar mechanisms. Huang et al. [[10]] released 5 of the earliest confirmed 41 patients with new-type coronavirus pneumonia in Wuhan (12%) diagnosed with virus-associated heart injury, which was mainly manifested by increased hs-cTnI levels (> 28 pg/mL); 4 out of 5 people received ICU, accounting for 31% of the total number of ICU patients. Hou Tao’s analysis of 84 patients with novel coronavirus pneumonia from January 1, 2020, to January 22, 2020, also pointed out that myocardial enzymes increased during treatment, especially myocardial kinase (CK), and the increase of myocardial kinase isoenzyme (CKMB), suggesting that the patient’s condition is serious and predicting that the patient’s condition is worsening. The recently released “Pneumonitis Diagnosis and Treatment Program for New Coronavirus Infection (Trial Fifth Edition)” pointed out that troponin increased in some newly critical patients. Although the current number of cardiovascular symptoms as the main manifestation is relatively small, from the current data, once the heart is involved, most patients are severely symptomatic. Its mechanism and processing method need to be further studied. How to antagonize COVID-19 on ACE2-mediated myocardial cells and microenvironment is the key to improve myocardial injury. With the increase in the number of cases of COVID-19 pneumonia, the number of patients with heart injury cannot be ignored, and it needs to be detected and treated in time. Myocardial histopathology and changes in ACE2-related pathways can help us understand its mechanism and evaluate clinical treatment effects.

### Respiratory system injury

As the lung is the major target organ of COVID-19 infection, initial onset of respiratory symptoms is common among patients. Chen et al. [11] retrospectively analyzed the clinical data from 99 patients with COVID-19 acute respiratory disease and found that 76% of patients received oxygen therapy and 17% received mechanical ventilation (of which 13% were non-invasive and 4% were invasive). Seventeen patients developed acute respiratory distress syndrome (ARDS). About 75% of cases who received chest computed tomography manifested as bilateral pneumonia with patterns of small leafy high-density patches and ground glass shadows. ACE2 is not only the gateway of COVID-19 in the lung but also probably involved in the development of lung injury. Research results by ZUO et al. [12] showed that ACE2 protein is mainly expressed in 1.4% of type II alveolar epithelial cells and barely present in other lung cells, such as type I alveolar epithelial cells, bronchial epithelial cells, endothelial cells, fibroblasts, and macrophages. Research results by Kubal et al. [13] and Imai et al. [14] showed that blocking the renin-angiotensin signal pathway can alleviate severe acute lung injury caused by SARS-CoV spike protein, suggesting that renin-angiotensin systems (RAS), composed of ACE, angiotensin II, and type 1a angiotensin II receptor (AT1a), promote the pathogenesis of the disease, induce pulmonary edema, and impair lung function. Hence, ACE2 plays a role in improving severe pulmonary edema and acute lung failure as the counter regulator of the RAS. In addition, the deletion of the ACE2 gene also promotes TGF-β/Smad signaling pathway-mediated tissue fibrosis and NF-κB-mediated inflammation. The combination of the coronavirus S protein and ACE2 downregulates the levels of ACE2 in the lungs; AngII levels rise; AT1 receptors are overactivated; the renin-angiotensin system is imbalanced in the lungs, which leads to acute lung injury such as pulmonary edema symptoms [15]. ACE2 plays a key role in the pathogenesis of acute lung injury, and it is inferred that it is also critical in COVID-19-induced lung injury. Although there are opinions that ACE2 plays a protective role in ALI (acute lung injury), the lack of ACE2 in the lung may be one of the causes of ALI, but the mechanism is still not completely clear. The current research focuses on ACE2 enzyme activity and substrate AngII and its catalytic products Ang-(1-7 [16]. COVID-19 has invaded the body through ACE2 and caused severe lung injury. Exploring the pathophysiological mechanism downstream of ACE2 during the inflammatory storm is the key to solving the problem. Etiology, pathophysiology, and evaluation of therapeutic effects require extensive histopathological research. How to maintain the integrity of the alveolar interstitial between the alveoli and the alveoli, especially the integrity of the microvascular structure between the alveoli, is of great significance for the relief of interstitial exudation, and the role of Chinese medicine in it is worth exploring. The evaluation of relevant effective or harmful treatment effects needs to be confirmed based on the statistical results of relevant big data.

### The intestinal damage

In addition to fever and cough, the most frequent symptoms of COVID-19 infection, some cases may progress to gastrointestinal symptoms, such as nausea, vomiting, and diarrhea, that are more severe in patients infected with COVID-19 than SARS-CoV and MERS-CoV. Some scholars in the USA reported on a patient who had diarrhea and abdominal discomfort in addition to persistent fever and dry cough during his hospitalization. It is worth mentioning that a new coronavirus (rRT-PCR positive result) was also detected in stool samples of diarrhea [17]. Genetic analysis by Zhang et al. [18] demonstrated high levels of COVID-19 receptor ACE2 expression in esophageal stratified epithelial cells, and ileocolic absorbable epithelial cells suggested a potential route of transmission via gastrointestinal system. ACE2/Ang-(1-7) axis is known as the main peptide on counteracting AngII effects and reducing inflammation. COVID-19 entry may be associated with ACE2 abnormal expression and dysfunction, leading to intestinal inflammation. Hashimoto et al. [19] found severe colon ulcer injury in mice in DSS-induced colitis mice with ACE2 gene knockout, and the concentration of AngII significantly increased in colon tissues. After treatment with recombinant soluble ACE2 (rsACE2), AngII concentration in the colon of DSS-treated ACE2 mutant mice was reduced to initial levels. Khajah et al. [20] found that DSS-induced colitis mice increased the expression of AngII and ACE2 in colonic mucosa and the expression of Ang-(1-7) also increased significantly. The expression of AngII decreased and the phosphorylation of p38, ERK1/2, and Akt signaling pathways were significantly inhibited. At the histological level, colonic mucosal damage and ulcers improved. Ang-(1-7) can also reduce intestine inflammation by directly inhibiting the activation of signal pathways such as ERK1/2 and NFκB via the Mas receptor. Therefore, gastrointestinal symptoms of COVID-19 infection may be related to invasion of intestinal epithelial cells expressing ACE2. The intestine is a possible target organ for COVID-19 infection, but whether digestive system is a route of transmission requires much further study. How to regulate the ACE2/Ang-(1-7)/Mas receptor axis, like supplying a therapeutically effective amount Ang-(1-7) to patients, need to be further explore.

### Abnormal coagulation system

Studies have found that some patients with new type of coronavirus pneumonia (NCP) have abnormal coagulation function and almost all critically ill patients have coagulation disorder [10, 11] In patients with novel coronavirus pneumonia (NCP), there may be multiple factors that promote thrombosis. On the one hand, the acute inflammatory response caused by severe infection or sepsis can affect the coagulation and fibrinolytic system in multiple ways. In addition, there is a certain correlation between ACE2 and coagulation. Fraga-Silva et al. [21] induced thrombosis in the vena cava of spontaneously hypertensive rats (SHR) and Wistar Kyoto rats (WKY) and measured ACE2 and ACE activity in the thrombus. Use of ACE2 inhibition (DX600; 0.1 mmol/L/kg) increased thrombus weight by 30%, and XNT (new ACE2 activator) treatment (10 mg/kg) reduced thrombus formation in SHR by 30%. In addition, XNT reduced platelet attachment to damaged blood vessels, reduced thrombus size, and prolonged the time to complete occlusion of blood vessels in mice. Therefore, a decrease in thrombotic ACE2 activity is associated with an increase in thrombosis in SHR. In addition, the activation of ACE2 can reduce the formation of thrombus and adhesion of platelets to blood vessels. In patients with novel coronavirus pneumonia (NCP), the abnormalities in the coagulation system can be seen, mostly in a hypercoagulable state, which can easily induce the formation of thrombus. There may be local embolism in the small vessels and microvessels of the relevant target organs. In addition, some critically ill patients with highly D-dimer and sudden death may shed the deep vein thrombus and aortic embolism, which will cause the condition to worsen sharply. Therefore, the abnormal concentration and activity of ACE2 may affect the coagulation system. Whether the physiological process of the disease exists requires relevant signal pathways and histopathological verification. For high-risk patients, an early assessment of thrombotic risk is required. In addition to traditional testing items, the detection of ACE2 may also be helpful in identifying patients with high thrombotic risk. Patients with a tendency to hypercoagulability may benefit from appropriate early anticoagulation and microcirculatory spasm treatment. For improving microcirculation, Chinese patent medicines with blood circulation and blood stasis removal, such as angelica, safflower, and salvia miltiorrhiza, may be effective for COVID-19 patients.

### Kidney, testicular, and liver damage

A study [22] analyzed the data of 59 cases in several hospitals, including 28 severe cases, to investigate their renal function between January 21 and February 7, 2020, situation. Of these, 63% (32/51) of patients showed proteinuria, suggesting renal insufficiency. Plasma creatinine and urea nitrogen increased in 19% (11/59) and 27% (16/59) of patients, respectively. Computed tomography (CT) scans revealed abnormal kidney imaging in 100% (27/27) of patients. Some scholars have stated that the expression level of ACE2 protein in the kidney, especially renal tubular cells, is significantly higher but the mRNA expression level is not high; ACE2 expression is not observed in immune cells and glomerular wall epithelial cells [23]. Renal tubular cells have reabsorption and excretion functions and play a key role in the excretion of metabolites, the maintenance of body fluid balance, and the acid-base balance. COVID-19 can enter renal tubular cells by binding to ACE2, causing cytotoxicity and abnormal renal function. Renal function tests and follow-up should be performed on neocoronavirus-infected patients to detect renal impairment in a timely manner and deal with it as early as possible. The study [23] also found that at the protein and mRNA levels, the expression of ACE2 in the testis is almost the highest in humans. Therefore, COVID-19 is likely to invade and damage the testicular tissue of patients by binding to these ACE2-positive cells. Therefore, clinicians should pay attention to the risk of testicular disease during hospitalization and later clinical follow-up, especially the assessment of fertility of young male patients and appropriate intervention. A retrospective study of 99 NCP patients from Wuhan [11] showed that 43 patients had varying degrees of abnormal liver function biochemical tests, of which 1 showed a significant increase in serum aminotransferase (ALT 7590 U/L, AST 1445 U/L). An increase in serum lactate dehydrogenase was observed in 75 patients, and an increase in serum creatine kinase was observed in 13 patients. The article did not further analyze whether these enzymatic abnormalities were COVID-19 infection itself or the liver damage caused by the drug used, nor did it describe the underlying status of the patient’s liver. Researchers [24] evaluated cell types that specifically expressed ACE2 in healthy liver tissue and found that bile duct cells highly expressed the neocoronavirus receptor ACE2, while hepatocytes expressed very low. These results indicate that liver damage in patients with new coronavirus pneumonia may be caused by the virus directly binding to ACE2-positive bile duct cells and causing bile duct dysfunction, or toxic side effects caused by therapeutic drugs, rather than the virus directly binding to liver cells. These results suggest that medical workers who are treating patients with neocoronavirus infection need to pay attention to patients’ liver reactions, especially those related to bile duct cell function, and need special care for patients with neocoronary pneumonia who have abnormal liver function.

## ACE2 is a potential therapeutic target

### Block virus intrusion

After SARS, many drugs were developed for the virus receptor ACE2, Han et al. [25] used alanine scanning mutagenesis to identify the key sites of ACE2 binding to SARS-CoV. The results showed that the charged amino acids between the 22nd and 57nd residues are important, especially K26 and D30. Researchers use these amino acids to artificially synthesize related peptides and evaluate their role in antivirals. The two peptides (aa22-44 and aa22-57; P4 and P5) showed moderate antiviral activity, and their half inhibitory concentrations (IC50) were about 50 μM and 6 μM, respectively. In addition, a peptide (P6 peptide) synthesized by artificially connecting two discontinuous segments (aa22-44 and aa351-357) in ACE2 with glycine showed strong antiviral activity, and its IC50 was about 0.1 μM. Huentelman et al. [26], based on the structure-based method, selected 140,000 small molecules through the docking of silicon molecules and selected molecules with high binding capacity to further determine the ACE2 enzyme inhibitory activity and the ability to inhibit SARS coronavirus S protein-mediated cell fusion. Studies have found a new human ACE2 inhibitor, NAAE. NAAE’s ability to regulate ACE2 activity and prevent SARS-S protein-mediated cell fusion indicates that it is a potentially valuable lead compound. ACE2 derivatives (P4, P5, and P6) or small molecules (NAAE) are currently on the market. They are effective in blocking SARS-CoV invasion. The receptors for COVID-19 are the same. Whether these drugs are effective or not remains to be confirmed. Studies [1] have confirmed the 5 key amino acids of SARS virus S protein interacting with ACE2, 4 of which have changed in COVID-19. S protein is also required for the development of the above drugs. The structure of these two viruses is different, which may affect the efficacy of the drug. However, using the same research method may develop effective targeted drugs as soon as possible.

### Inhibit inflammation and reduce the damage of target organs

Many targeted drugs are currently at the stage of clinical or even animal research. The main method is still symptomatic treatment, which can timely inhibit the occurrence and development of its inflammation and reduce the damage to the target organs by the virus, thereby improving the prognosis. The low expression of ACE2 caused by virus infection activates the renin-angiotensin system (RAS), which aggravates lung injury. [13]. Therefore, the activation of the ACE2-Ang-(1-7) -Mas receptor pathway or inhibition of the ACE-AngII-AT1R receptor pathway may benefit patients. In animal models, blocking angiotensin II receptor I (AT1R) can alleviate the lung injury mediated by SARS-CoV spike protein. Studies by Henry et al. [27] suggest that ACEI and statins may have a certain effect on patients with viral pneumonia who are not coronavirus infected and have no underlying disease. The use of ACEI in patients with novel coronavirus pneumonia is currently controversial. It can inhibit RAS and may play a role in protecting the lungs and controlling symptoms. Under normal physiological conditions, ACE2 and ACE are in equilibrium. The use of ACEI can inhibit ACE, leading to increased expression of ACE2, which in turn increases the risk of infection. Ferrario et al. [28] showed that the inhibitory effect of ACEI or AT1R blocker on RAS would increase the expression of ACE2 mRNA and ACE2 activity but did not increase the ACE2 concentration. In addition, the expression level of ACE2 is inconsistent with the virus attack. For example, ACE2 is highly expressed in the heart and kidney, but serious lesions are rare in these organs, and the mechanism is still unclear. It is possible that viral infection also requires other receptors or cofactors. Recently, in a phase II clinical trial of ARDS patients using recombinant human ACE2 (GSK2586881), this compound has been widely used in ARDS patients and can reduce AngII levels, increasing Ang-(1-7) and surfactant protein D level [29]. It should be studied that exogenous supplementation with ACE2 for COVID-19 patients.

### Chinese medicine treatment

Drugs that block virus invasion, such as P4 and P5, may have an impact on the physiology (blood pressure regulation) of the ACE2 receptor itself. Inhibition of classic RAS may reduce the inflammatory response, but at the same time, adverse reactions such as hyperkalemia, hypotension, and cough may limit the use of such drugs. Many Chinese medicines cannot only act on ACE2 to treat COVID-2019 but also reduce the incidence of adverse reactions to a certain extent. The researchers collected 405 traditional Chinese medicine ingredients, using ACE2 as the target protein, and molecularly docked the binding region with SARS-CoV. Screening found 46 active ingredients of traditional Chinese medicine that can act on the COVID-19 S protein binding region with human ACE2 and have high binding energy, which are mainly attributed to mulberry leaves, *Atractylodes chinensis*, *Fritillaria cirrhosa*, ginger, honeysuckle, forsythia, grass fruit, and other 7 traditional Chinese medicines [30, 31]. Astragaloside can activate the ACE2- Ang-(1-7)-Mas pathway; increase the levels of ACE2, Ang-(1-7), and Mas; and play a role in regulating lung function, thereby effectively inhibiting respiratory failure [32].The protective effect of ginsenoside Rg3 on the kidney is mainly through upregulating the expression of ACE2 in the kidney, thereby increasing the degradation of AngII, reducing the inflammation and oxidative stress mediated by AngII in the kidney, and reducing the pathological changes in the kidney [33]. Use modern technology to discover effective Chinese medicine ingredients. The effect may be comparable to that of western medicine, but the adverse reactions are relatively few.

3. To sum up [12].

The ACE2 is an important protective protein in the human body and is also a necessary receptor for COVID-19 infection to invade the human body. ACE2 is downregulated after a virus infection in humans, which reduces the degradation of AngII, which promotes the inflammatory response. It reduces the production of Ang-(1-7) which relaxes blood vessels, improves endothelial function, and reduces proliferation. The imbalance of AngII-AT1R/AT2R axis and ACE2-Ang(1–7)-Mas leads to target organ damage. ACE2 is widely distributed, so COVID-19 can affect a variety of organs and show a variety of clinical manifestations. Those with atypical symptoms should pay more attention. Heart damage, mostly in high-risk groups, is identified early and treated accordingly; gastrointestinal symptoms may be caused by damage to the digestive system. Pay attention to the possibility of fecal-oral transmission. The abnormal concentration and activity of ACE2 may affect the coagulation system. Whether the physiological process of the disease exists requires relevant signal pathways and histopathological verification. The ACE2 is a potential therapeutic target. The strategy shoud be studied that developing ACE2 targeted drugs to block virus to integrate with ACE2 according to its structure, explore some effectiveness drugs for activate the ACE2-Ang-(1-7)-Mas receptor pathway, or inhibiting ACE-AngII-AT1R receptor pathway to suppress inflammation and reduce target organ damage. The role of ACE2 in COVID-19 infection needs further study. This article takes COVID-19 and SARS-CoV to invade targets as the main line, focuses on describing possible target organ damage, provides corresponding guidance for clinical diagnosis and treatment, explains the possible role of ACE2 in different organ damage, and provides the basis for follow-up, and Clinical research provides possible directions and ideas; ACE2 is not only a “gateway” for virus invasion but also a key substance that causes organ damage. Finding possible treatment strategies starting from ACE2 has the effect of killing two birds with one stone and has broad application prospects and clinical value.
